# Aumolertinib plus chemotherapy as first-line treatment for advanced NSCLC with EGFR exon 19 deletion or exon 21 L858R: a phase II trial

**DOI:** 10.1093/oncolo/oyae336

**Published:** 2025-03-15

**Authors:** Yanwei Li, Chenguang Li, Xiaoliang Zhao, Yong Li, Feng He, Zhanyu Pan

**Affiliations:** Department of Integrated Traditional and Western Medicine, Tianjin Medical University Cancer Institute and Hospital, National Clinical Research Center for Cancer, Tianjin, People’sRepublic of China; Academy of Medical Engineering and Translational Medicine, Tianjin University, Tianjin 300192, People’s Republic of China; Tianjin’s Clinical Research Center for Cancer, Tianjin, People’s Republic of China; Key Laboratory of Cancer Prevention and Therapy, Tianjin, People’s Republic of China; Academy of Medical Engineering and Translational Medicine, Tianjin University, Tianjin 300192, People’s Republic of China; Academy of Medical Engineering and Translational Medicine, Tianjin University, Tianjin 300192, People’s Republic of China; Academy of Medical Engineering and Translational Medicine, Tianjin University, Tianjin 300192, People’s Republic of China; Department of Integrated Traditional and Western Medicine, Tianjin Medical University Cancer Institute and Hospital, National Clinical Research Center for Cancer, Tianjin, People’sRepublic of China; Tianjin’s Clinical Research Center for Cancer, Tianjin, People’s Republic of China; Key Laboratory of Cancer Prevention and Therapy, Tianjin, People’s Republic of China; Academy of Medical Engineering and Translational Medicine, Tianjin University, Tianjin 300192, People’s Republic of China

**Keywords:** non-small cell lung cancer, EGFR mutation, aumolertinib, platinum-based chemotherapy

## Abstract

**Background:**

To evaluate the efficacy and safety of aumolertinib combined with pemetrexed and carboplatin as first-line treatment in advanced non-small-cell lung cancer (NSCLC) patients with epidermal growth factor receptor (EGFR) mutation (exon 19 deletion or exon 21 L858R).

**Methods:**

In phase II trial (NCT04646824), patients received aumolertinib 110 mg once daily plus pemetrexed (500 mg/m^2^) and carboplatin (area under curve = 5) once every 3 weeks for 4 cycles, followed by maintenance aumolertinib (110 mg once daily) and pemetrexed (500 mg/m^2^ once every 4 weeks). The primary endpoint was progression-free survival (PFS). Secondary endpoints included objective response rate (ORR), disease control rate (DCR), overall survival (OS), and safety.

**Results:**

From November 2020 to October 2021, 34 patients were included for analysis. The median PFS was 28.0 months (95% CI, 18.7-36.9). The ORR was 91.2% (31/34), and the DCR was 100%. The median OS was not reached. Of 28 patients with circulating tumor DNA (ctDNA) testing, 22 (78.6%) showed clearance of EGFR mutation after 2 or 4 cycles. The median PFS was 31 months in patients with EGFR mutation clearance in ctDNA, and the ORR of them was higher than those without EGFR mutation clearance in ctDNA (90.9% vs 33.3%). The most common grade ≥ 3 treatment-related adverse event was decreased neutrophil count (22 [64.7%]).

**Conclusion:**

Aumolertinib plus chemotherapy shows potential as first-line treatment for patients with EGFR-mutant advanced NSCLC, which deserves to be investigated in randomized controlled trials. CtDNA clearance may be a prognostic marker.

**Implications for practice**

First-line aumolertinib plus pemetrexed and carboplatin shows promising objective response rate and progression-free survival benefit for patients with advanced non-small-cell lung cancer harboring epidermal growth factor receptor exon 19 deletion or exon 21 L858R, with an acceptable safety profile. Circulating tumor DNA clearance may be a potential prognostic marker.

## Introduction

Lung cancer is a prevalent and lethal malignancy worldwide,^1^ with non-small-cell lung cancer (NSCLC) being the predominant subtype. Epidermal growth factor receptor (EGFR) mutation is one of the most common oncogenic mutations in NSCLC.^2^ Based on the phase III FLAURA study,^3^ third-generation EGFR tyrosine kinase inhibitor (TKI) osimertinib has been recommended as preferred first-line treatment for patients with EGFR-mutant advanced NSCLC. However, acquired drug resistance remains a clinical challenge for patients with EGFR mutations, even after the emergence of third-generation EGFR-TKIs.^4,5^ Combinations of different drugs that can delay or reverse drug resistance are urgently needed.

It is demonstrated that the addition of chemotherapy can extend the survival benefit of EGFR-TKIs. First-generation EGFR-TKI gefitinib combined with chemotherapy significantly improved progression-free survival (PFS) and overall survival (OS) compared with gefitinib monotherapy, but with increased toxicities.^6,7^ In the phase III FLAURA2 study, the addition of chemotherapy to osimertinib significantly prolonged the median PFS from 19.9 months to 29.4 months (hazard ratio, 0.62; 95% CI [CI], 0.48-0.80), as assessed by blinded independent central review, and the toxicity profile of combination therapy was consent with the known profile of individual drugs.^8^ Third-generation EGFR-TKI combined with chemotherapy may be a novel standard or care for patients with EGFR-mutant advanced NSCLC in the first-line setting.

Aumolertinib (formerly almonertinib; HS-10296) is an oral, third-generation EGFR-TKI that has been approved for the first-line treatment of patients with EGFR-mutant advanced NSCLC in China. The phase III AENEAS study showed a significantly better median PFS with aumolertinib than with gefitinib (19.3 months vs 9.9 months; hazard ratio, 0.46; 95% CI, 0.36-0.60), as determined by investigator assessment.^9^ Here we conducted this study to investigate the efficacy and safety of aumolertinib combined with chemotherapy as first-line treatment for patients with EGFR-mutant advanced NSCLC.

## Patients and Methods

### Study design and participants

This was a single-center, single-arm, phase II trial conducted at Tianjin Medical University Cancer Institute and Hospital in China. Eligible patients met the following criteria: (1) aged 18 years or older; (2) histologically or cytologically confirmed NSCLC or clinically diagnosed advanced peripheral lung cancer with positive plasma results of EGFR-sensitizing mutation (exon 19 deletion or exon 21 L858R); (3) no prior systemic therapy in advanced setting; (4) an Eastern Cooperative Oncology Group (ECOG) performance status of 0-2; (5) at least one measurable lesion according to the Response Evaluation Criteria In Solid Tumors (RECIST), version 1.1^10^; (6) expected survival of at least 3 months; and (7) adequate bone marrow, liver and kidney function. Full inclusion and exclusion criteria are available in the trial protocol.

The study was conducted in accordance with the principles of the Declaration of Helsinki and approved by the ethics committee of Tianjin Medical University Cancer Institute and Hospital. Written informed consent was obtained from each patient before enrollment. The study was registered with ClinicalTrials.gov (NCT04646824).

### Procedures

Patients were treated with oral aumolertinib 110 mg once daily plus intravenous pemetrexed (500 mg/m^2^) and carboplatin (area under curve [AUC] = 5) once every 3 weeks for 4 cycles, followed by maintenance aumolertinib (110 mg once daily) and pemetrexed (500 mg/m^2^ once every 4 weeks) until disease progression, withdrawal of consent, or unacceptable toxicity. Patients with progression after first-line aumolertinib plus chemotherapy received second-line treatment per best local standard of care.

Clinical assessment was performed prior to every treatment cycle. Radiographic response was evaluated by investigators using contrast-enhanced computed tomography per RECIST 1.1 every 2-3 months.^10^

### Outcomes

The primary endpoint was PFS, defined as the time from the commencement of treatment to either documented disease progression or death from any cause. Secondary endpoints included ORR (proportion of patients with the best overall response of complete or partial response), disease control rate (DCR; proportion of patients with the best overall response of complete response, partial response, or stable disease), OS (time from the commencement of treatment to death from any cause), and safety. Adverse events (AEs) were closely monitored throughout the study period and graded according to the National Cancer Institute Common Terminology Criteria for Adverse Events, version 5.0.

The exploratory endpoint was quality of life, evaluated using the European Organization for Research and Treatment of Cancer Quality of Life Questionnaire-Core 30 (EORTC QLQ-C30^11^) at baseline, 3 months, and 6 months. For functional subscales and global health status, higher scores indicate better function. For symptom subscales, higher scores indicate worse symptoms.

Post hoc analysis on the clearance of EGFR mutation in circulating tumor DNA (ctDNA) was performed using plasma samples by the Super-ARMS EGFR Mutation Detection Kit (AmoyDx). Tumor tissue samples were collected by biopsy. Programmed cell death-ligand 1 (PD-L1) expression measurement, ctDNA testing, and next-generation sequencing were performed by Burning Rock Biotech Ltd (Guangzhou, China). PD-L1 positivity was defined as tumor proportion score (TPS) ≥ 1%.

### Statistical analysis

Sample size was calculated based on the single-sample log-rank test using PASS 15.0. The null hypothesis of median PFS was 18.9 months with third-generation TKI alone.^9^ The alternative hypothesis of median PFS was 24.9 months with aumolertinib plus chemotherapy. The enrollment was expected to last 12 months and the follow-up was expected to last 24 months. With a significance level of 0.05, power of 80%, and a drop-out rate of 10%, a total of 42 patients were required.

Continuous variables were expressed as mean ± standard deviation or median (range) as appropriate. Categorical variables were expressed as frequency and percentage. Survival was estimated using the Kaplan-Meier method. ORR was compared using Fisher’s exact test. Comparisons of QLQ-C30 scores between baseline and 3 or 6 months were performed using the paired *t* test. A post hoc analysis of second PFS (PFS2; time from the first progression to either second documented disease progression or death from any cause) was performed in patients who received second-line treatment after progression on first-line aumolertinib plus chemotherapy. Statistical analyses were performed using SPSS 20.0. *P* ≤ 0.05 (unless otherwise specified) was considered statistically significant.

## Results

### Patient characteristics

**Figure 1.**
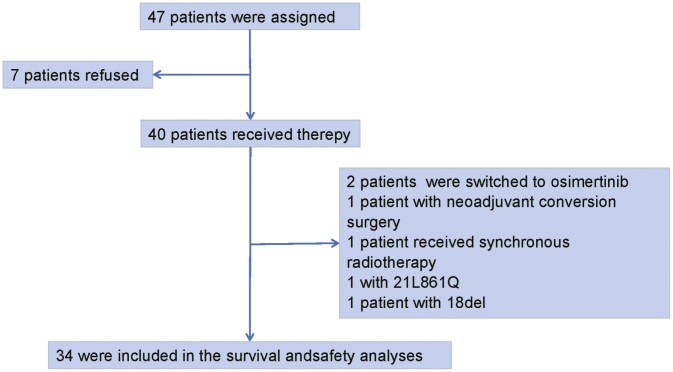
Patient flowchart.

From November 7, 2020 to October 9, 2021, a total of 40 patients received the study treatment. Four patients also received other anti-cancer treatments during the study period, and 2 patients were found not meeting inclusion criteria after the initiation of the study treatment. These 6 patients were excluded from the efficacy and safety analyses (Figure 1). Baseline characteristics of the remaining 34 patients are shown in Supplementary Table S1. The median age was 59 years (range, 37-82). One (2.9%) patient had stage IIIC disease, and 33 (97.1%) had stage IV disease. Twenty (58.8%) patients had EGFR exon 19 deletion, and 14 (41.2%) had EGFR exon 21 L858R. One (2.9%) of these 34 patients also had primary EGFR 19 T790M mutation, and 13 (38.2%) patients also had TP53 mutation. Seventeen (50.0%) patients had ECOG performance status of 2. Nineteen (55.9%) patients had brain metastases at baseline, including 17 (50.0%) patients with brain parenchymal metastases only, one (2.9%) with meningeal metastases only, and one (2.9%) with brain parenchymal and meningeal metastases. All the brain metastases were untreated, and 2 (5.9%) patients had symptomatic brain metastases. Twenty-six (76.5%) patients had PD-L1-positive disease, including 19 (55.9%) patients with PD-L1 high expression (TPS > 50%).

### Treatment

**Figure 2.**
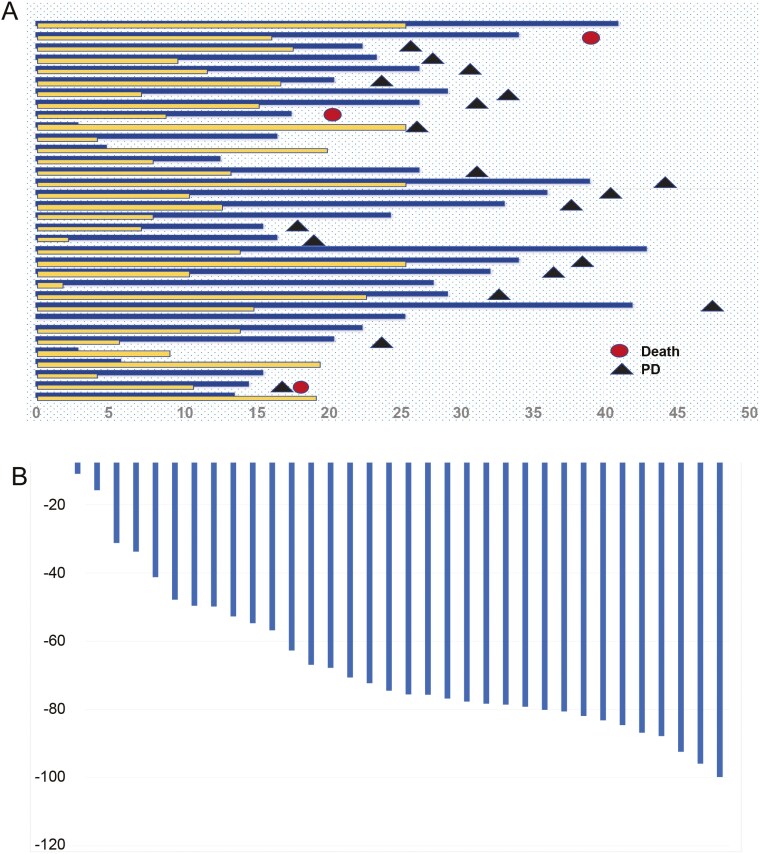
(A) Swimmer plot. (B) Waterfall plot.

By the data cutoff date on November 10, 2023, the median follow-up duration was 30 months (range 13-38). Thirty-two (94.1%) of 34 patients completed the initial 4 cycles of aumolertinib plus chemotherapy and received maintenance aumolertinib and pemetrexed. One of the remaining 2 patients, an 82-year-old patient, received only one treatment cycle due to intolerance, while the other patient received 2 cycles but declined to continue initial treatment due to complete remission of symptoms. These 2 patients received maintenance aumolertinib. For all 34 patients, the median number of chemotherapy cycles was 20.5 (range 1-35; Figure 2A). The median duration of aumolertinib treatment was 26.1 months (range 14.3-34.3).

Sixteen of 19 patients with progression after first-line aumolertinib plus chemotherapy received second-line bevacizumab plus paclitaxel (paclitaxel liposomes or albumin-bound paclitaxel) and carboplatin; 10 patients completed the 4 cycles of second-line bevacizumab plus chemotherapy, and 15 patients received maintenance bevacizumab. The other 3 of 19 patients with progression after first-line aumolertinib plus chemotherapy received individualized second-line treatment. One patient with EGFR C797S mutation received first-generation TKI, with a PFS2 of 4 months. One patient with small cell transformation received carboplatin plus etoposide, with a PFS2 of 3 months. One patient with c-Met amplification received crizotinib plus osimertinib, with a PFS2 of 5 months.

### Radiographic response

Of 34 patients, 31 patients achieved partial response and 3 had stable disease (Figure 2B), with an ORR of 91.2% and a DCR of 100%. Subgroup analyses showed that the ORR was 94.7% in patients with exon 19 deletion, 90.9% in patients with exon 21 L858R, 88.2% in patients with baseline brain metastases, and 100% in patients with TP53 mutation.

**Figure 3.**
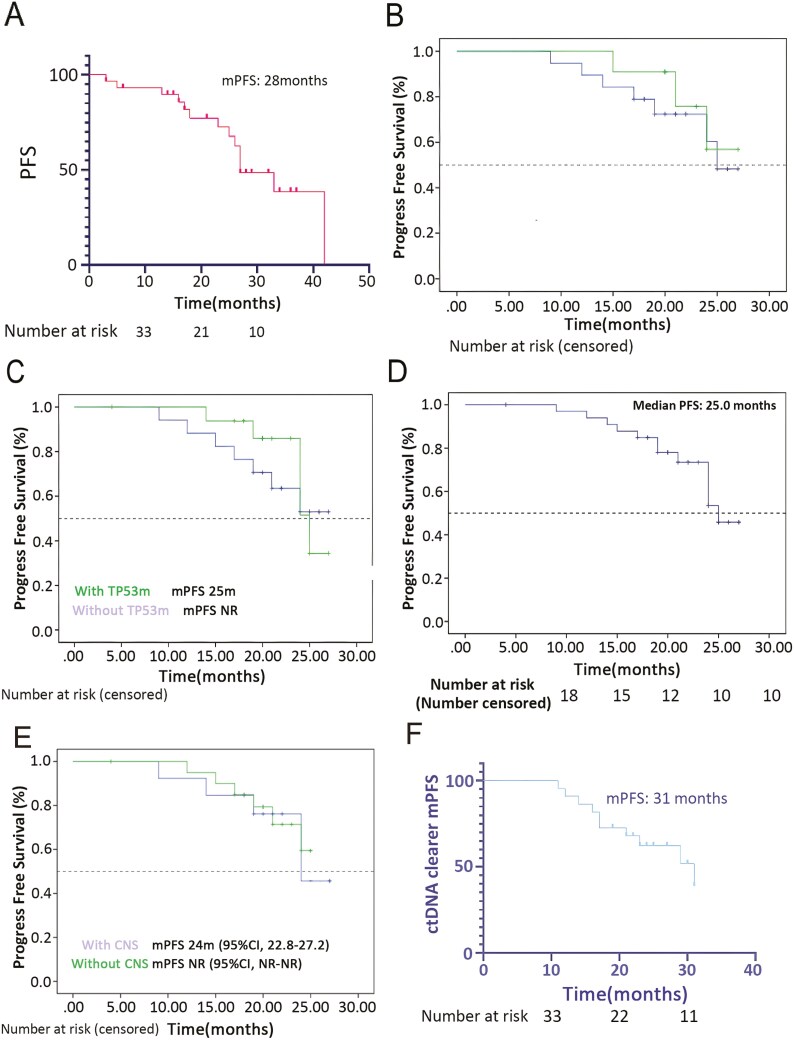
Kaplan-Meier curves of PFS. (A) PFS in all patients. (B) PFS in patients with exon 19 deletion or exon 21 L858R. (C) PFS in patients with or without TP53 mutation. (D) PFS in patients with PD-L1 > 50%. (E) PFS in patients with or without baseline brain metastases. (F) PFS in patients with EGFR mutation clearance in circulating tumor DNA.

Survival of 34 patients, 17 patients had radiographic progression (including six intracranial progression), 2 had symptom deterioration, and 2 died. The estimated median PFS was 28.0 months (95% CI, 18.7-36.9; Figure 3A) in 34 patients. The PFS rate was 94%, 71%, and 35% at 1, 2, and 3 years, respectively. Subgroup analyses showed that the median PFS was 25.0 months (95% CI, 15.3 to 37.6) in patients with exon 21 L858R, NR (95% CI, NR to NR) in patients with exon 19 deletion 25.0 months (95% CI, 20.6-29.4) in patients with TP53 mutation, 25.0 months (95% CI, 19.3-26.8) in patients with PD-L1 > 50%, and 25.0 months (95% CI, 17.4-28.4) in patients with baseline brain metastases (Figure 3B-E).

For the 16 patients who received second-line bevacizumab plus paclitaxel and carboplatin after progression on first-line aumolertinib plus chemotherapy, the median PFS2 was 10.0 months (95% CI, 6.4-12.7).

The median OS for 34 patients was NR, with 14 death events by the data cutoff date.

### Safety

Treatment-related AEs (TRAEs) are listed in Supplementary Table S2. The most common any grade TRAEs were decreased neutrophil count (26 [76.5%]), fatigue (21 [61.8%]), elevated aminotransferase (17 [50.0%]), anorexia (13 [38.2%]), and anemia (12 [35.3%]). The most common TRAEs of grade 3 or worse were decreased neutrophil count (22 [64.7%]), fatigue (6 [17.6%]), and anemia (6 [17.6%]).

Owing to AEs, 2 (5.9%) patients had dose reduction of aumolertinib, and no patients interrupted or discontinued aumolertinib. Three (8.8%) patients had dose reduction of pemetrexed, one (2.9%) interrupted pemetrexed, and none discontinued pemetrexed. Dose reduction, interruption, and discontinuation of carboplatin occurred in 16 (47.1%), 7 (20.6%), and 3 (8.8%) patients, respectively.

### EGFR mutation clearance in ctDNA

**Figure 4.**
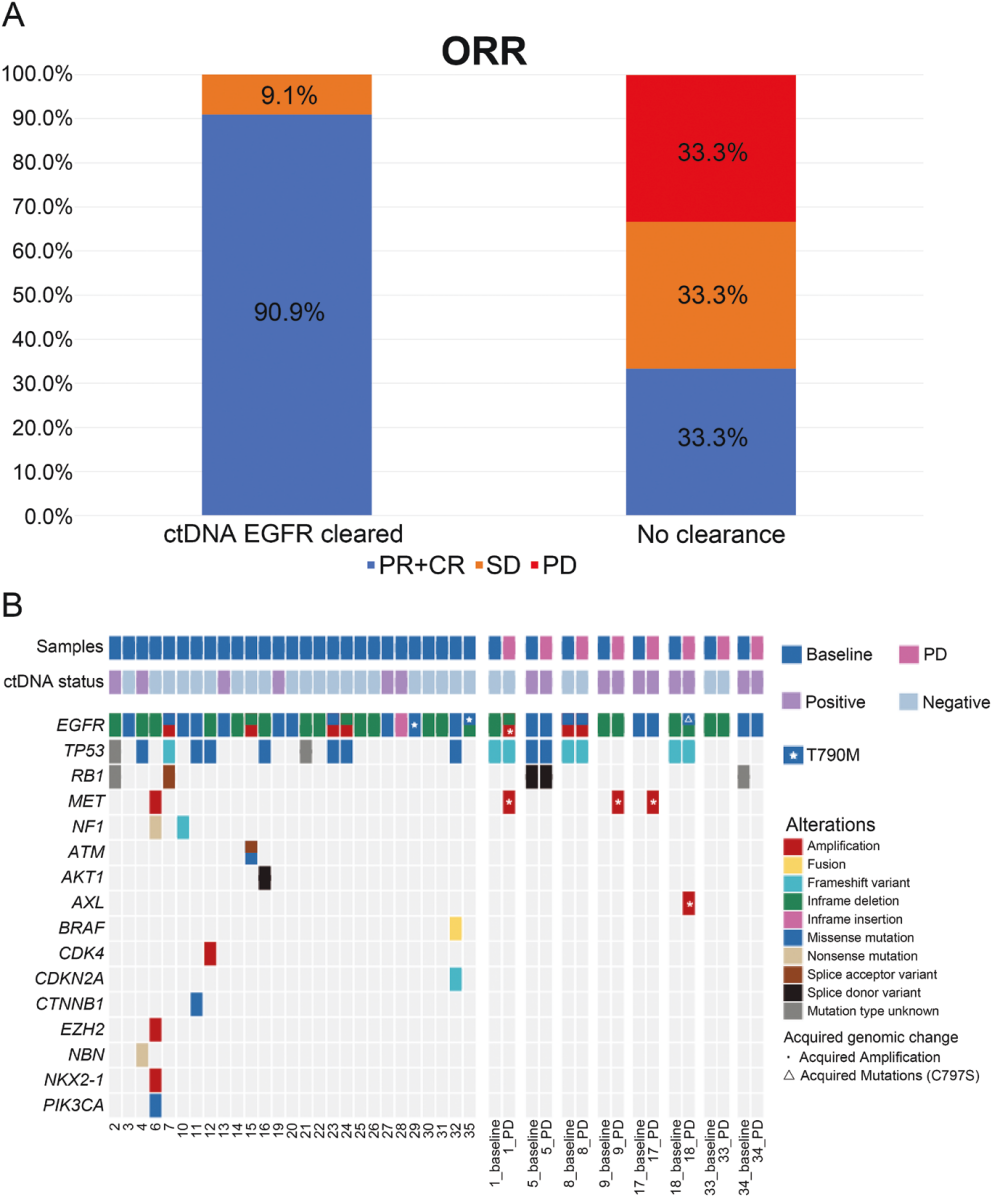
(A) Tumor response in patients with or without EGFR mutation clearance in circulating tumor DNA. (B) Resistance pattern at progression on aumolertinib plus pemetrexed and carboplatin.

Twenty-eight patients had detectable ctDNA at baseline, of whom 22 (78.6%) showed clearance of EGFR mutation in ctDNA after 2 or 4 cycles of treatment. The ORR of patients with EGFR mutation clearance was significantly higher than that of those without EGFR mutation clearance (90.9% vs 33.3%, *P* = .001; Figure 4A). The median PFS was 31 months (95% CI, 21.0-41.0) in patients with EGFR mutation clearance (Figure 3F), and 25.0 months (95%CI, 14.3-34.3) in those without EGFR mutation clearance.

### Resistance pattern

Of 8 patients who underwent pre/post progression resistance testing with tissue biopsy, 4 were found to have alterations known to represent putative resistance mechanisms to third-generation EGFR TKIs, including 2 with MET amplification, one with C797S mutation and AXL amplification, and one with EGFR and AXL amplifications (Figure 4B).

### Quality of life evaluation

**Figure 5.**
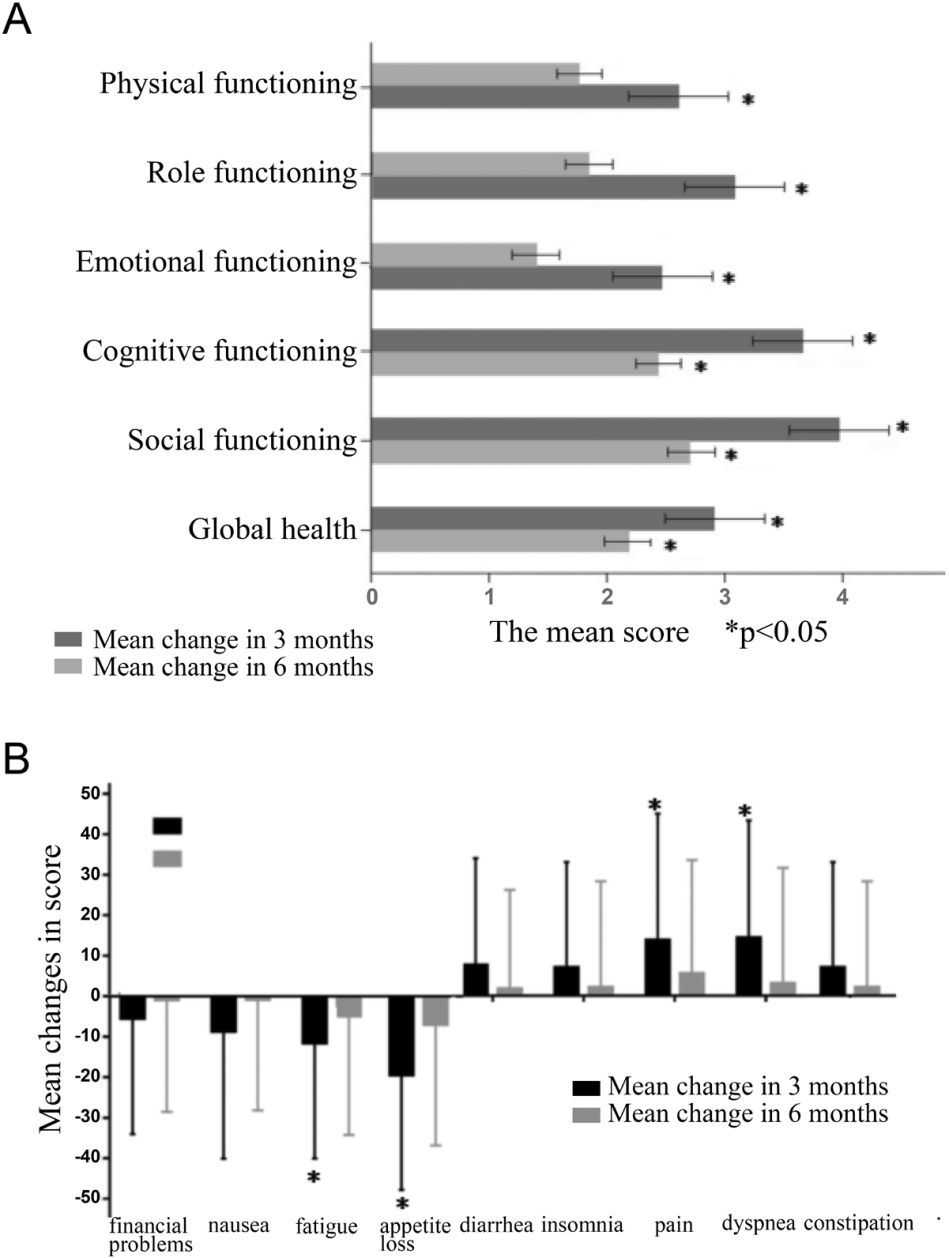
Changes in European Organization for Research and Treatment of Cancer Quality of Life Questionnaire-Core 30 scores from baseline to 3 and 6 months. Asterisk indicates the significant difference in score between baseline and the corresponding time point (*P* < .05).

The completion rate of QLQ-C30 questionnaire was 94.1% (32/34) at all 3 time points (baseline, 3 months, and 6 months). Mean scores of physical, role, emotional, cognitive and social functioning, and global health status were significantly increased after 6 months compared with baseline (Figure 5A). Fatigue and appetite loss were significantly improved after 3 months but returned to baseline level after 6 months. Pain and dyspnea were significantly deteriorated after 3 months but returned to baseline level after 6 months. Constipation, insomnia, nausea, diarrhea, and financial problems remained unchanged after 3 and 6 months compared with baseline (Figure 5B).

## Discussion

Given the availability of multiple first-line treatment options with comparable PFS benefit for patients with EGFR-mutant advanced NSCLC, the sequencing of treatment is of importance. Of note, it is imperative to acknowledge that nearly 40% of the patients who discontinued first- to third-generation EGFR-TKI did not receive any subsequent treatment, underscoring the need for optimal upfront therapy.^12^ In light of these considerations, third-generation EGFR-TKI combined with chemotherapy may be a novel first-line therapy option, which has been demonstrated to improve ORR and survival compared with EGFR-TKI alone.^8^ Furthermore, the combination of third-generation EGFR-TKI and chemotherapy has similar safety profile with each individual drug.^8,13^ Our study supplements the first-line evidence of third-generation EGFR-TKI combined with chemotherapy for advanced NSCLC with EGFR exon 19 deletion or exon 21 L858R. The median PFS was 28.0 months with first-line aumolertinib plus pemetrexed and carboplatin. The ORR was 91.2%. The safety profile was acceptable. All these results suggest the promising value of aumolertinib plus chemotherapy for EGFR-mutant advanced NSCLC in the first-line setting.

As another third-generation EGFR-TKI, aumolertinib showed similar efficacy as osimertinib when used as first-line monotherapy, with a median investigator-assessed PFS of 19.3 months in AENEAS and 18.9 months in FLAURA.^3,9^ When combined with chemotherapy, our study also showed similar median investigator-assessed PFS (28.0 months vs 25.5 months) to that in FLAURA2.^8^ It should be noted that at least 50% of patients in our study had an ECOG performance status of 2 and/or brain metastases, which are exclusion criteria in most clinical trials. Even under this circumstance, the median PFS reached 28.0 months. This suggests the potential of aumolertinib plus chemotherapy as first-line treatment for patients with EGFR-mutant advanced NSCLC, including those with poor general condition, severe symptoms, or poor access to osimertinib.

Our study explored several predictive biomarkers to help target patients who are most likely to benefit from aumolertinib plus chemotherapy. In the phase III ADJUVANT/CTONG1104 study, 5 predictive biomarkers were identified (eg, TP53 exon 4/5 missense mutations and RB1 alterations).^14,15^ In our study, patients with TP53 mutation also exhibited numerically better therapeutic benefits from combination therapy than those without TP53 mutation. CtDNA clearance has been reported to be an early predictor of clinical response and PFS in patients treated with EGFR-TKIs.^16,17^ Our results showed that patients with clearance of EGFR mutation in ctDNA had a median PFS of 31 months. Due to the small sample size in our study, the predictive roles of TP53 mutation and ctDNA clearance still require further investigations.

The phase III ATTLAS and ORIENT-31 studies consistently confirmed that patients with NSCLC who progressed on EGFR-TKI therapy could achieve PFS benefit with immune checkpoint inhibitor plus bevacizumab and chemotherapy compared with chemotherapy alone, and the median PFS was 8.7 months with atezolizumab plus bevacizumab and chemotherapy and 7.2 months with sintilimab plus IBI305 (bevacizumab biosimilar) and chemotherapy.^18,19^ However, no OS benefit was observed for these combinations over chemotherapy alone.^18,19^ The high incidence of grade 3 or worse TRAEs (35-56%) with the 4-drug combination regimen also raises concern.^18-20^ The role of this combination strategy in the second-line setting remains uncertain. The phase III KEYNOTE-789 study failed to prove the PFS or OS benefit with the addition of pembrolizumab to platinum-based doublet chemotherapy in patients with TKI-resistant, EGFR-mutant, metastatic non-squamous NSCLC.^21^ Our post hoc analysis complements the 3-drug evidence of bevacizumab combined with platinum-based doublet chemotherapy, with a median PFS2 of 10.0 months after progression on first-line aumolertinib plus chemotherapy. This combination showed a PFS benefit comparable to immune checkpoint inhibitor plus bevacizumab and chemotherapy while potentially sparing the toxicity of immune checkpoint inhibitor (which remains of unclear benefit). Bevacizumab plus chemotherapy may be a feasible option after resistance to EGFR-TKI. However, due to the small sample size, these results should be interpreted with cautions. In addition, re-exposure to platinum-based doublet chemotherapy may be not appropriate for all patients at progression, especially for those with limited performance status.

The impact of aumolertinib plus chemotherapy on quality of life was generally acceptable. Patients’ physical, psychological, and social functioning were improved during the treatment period, with most symptoms remaining stable. The overall toxicity of this combination therapy was manageable. No new safety signals were identified. However, from the perspective of clinical practice, there are still some concerns. The addition of chemotherapy can lead to increased safety risk, especially for hematologic toxicities. Compared with oral EGFR-TKI monotherapy, there may also be logistical and financial burdens when chemotherapy is added. In addition, our quality-of-life results showed that fatigue and appetite loss were significantly improved after 3 months of treatment but returned to baseline level after 6 months, which might be due to the cumulative impact of chemotherapy. For these reasons, first-line EGFR-TKI monotherapy may remain the standard of care for elderly or frail patients, while third-generation EGFR-TKI plus chemotherapy can be an alternative option for patients with good physical condition but high-risk disease.

Our study has some limitations. This was a single-center, single-arm study with a small sample size, thus the potential bias was inevitable. The median OS was NR due to inadequate follow-up. In the future, large-scale randomized controlled trials are warranted to validate our findings. In addition, oral agents as chemotherapeutic partners of aumolertinib will be explored.

## Conclusion

First-line aumolertinib plus pemetrexed and carboplatin shows promising ORR and PFS benefit for patients with advanced NSCLC harboring EGFR exon 19 deletion or exon 21 L858R, with an acceptable safety profile. CtDNA clearance may be a potential prognostic marker. Further randomized controlled trial is warranted.

## Supplementary Material

oyae336_suppl_Supplementary_Tables_1-2

## Data Availability

The data generated in this study are available upon request from the corresponding author.
