# Supplementary material for: Aumolertinib plus chemotherapy as first-line treatment for advanced NSCLC with EGFR exon 19 deletion or exon 21 L858R: a phase II trial
Source: Oncologist. 2025 Mar 15;30(3):oyae336. doi: 10.1093/oncolo/oyae336 (PMC11909725; doi:10.1093/oncolo/oyae336)
Supplement: oyae336_suppl_Supplementary_Tables_1-2 [file oyae336_suppl_supplementary_tables_1-2.docx]

**Supplementary Table 1** Baseline characteristics

| **Characteristics** | | Patients (n=34) |
| --- | --- | --- |
| Sex, n (%) | Male | 13 (38.2) |
|  | Female | 21 (61.8) |
| Age (years) | Median (range) | 59 (37-82) |
| Histology, n (%) | Adenocarcinoma | 34 (100) |
| Stage, n (%) | IIIC | 1 (2.9) |
|  | IV | 33 (97.1) |
| EGFR mutation, n (%) | Exon 19 deletion | 20 (58.8) |
|  | Exon 21 L858R | 14 (41.2) |
| TP53 mutation, n (%) | Yes | 13 (38.2) |
|  | No | 21 (61.8) |
| Smoking history, n (%) | Never | 28 (82.4) |
|  | Former | 5 (14.7) |
|  | Current | 1 (2.9) |
| ECOG performance status, n (%) | 0 | 8 (23.5) |
|  | 1 | 9 (26.5) |
|  | 2 | 17 (50.0) |
| Medical history, n (%) | None | 3 (8.8) |
|  | Hypertension | 12 (35.3) |
|  | Diabetes mellitus | 10 (29.4) |
|  | COPD or emphysema | 4 (11.8) |
|  | Prior tuberculosis | 1 (2.9) |
|  | Others | 21 (61.8) |
| Prior anti-cancer therapy, n (%) | Surgery with or without chemotherapy and/or radiotherapy | 5 (14.7) |
|  | Radical radiotherapy with or without chemotherapy | 3 (8.8) |
| Distant metastases, n (%) | Liver | 4 (11.8) |
|  | Lung | 12 (35.3) |
|  | Bone | 17 (50.0) |
|  | Brain | 19 (55.9) |
|  | Pulmonary embolism | 11 (32.4) |
| PD-L1 TPS, n (%) | >50% | 19 (55.9) |
|  | 1%-50% | 7 (20.6) |
|  | <1% | 8 (23.5) |

EGFR, epidermal growth factor receptor; ECOG, Eastern Cooperative Oncology Group; COPD, chronic obstructive pulmonary disease; PD-L1, programmed cell death-ligand 1; TPS, tumor proportion score.

**Supplementary Table 2** Treatment-related adverse events (n=34)

| **Events, n (%)** | **Any grade** | **Grade ≥3** |
| --- | --- | --- |
| Neutrophil count decreased | 26 (76.5) | 22 (64.7) |
| Fatigue | 21 (61.8) | 6 (17.6) |
| Elevated aminotransferase | 17 (50.0) | 0 (0) |
| Anorexia | 13 (38.2) | 1 (2.9) |
| Anemia | 12 (35.3) | 6 (17.6) |
| Skin fissures | 9 (26.5) | 0 (0) |
| Nausea and/or vomiting | 9 (26.5) | 1 (2.9) |
| Pruritus | 9 (26.5) | 0 (0) |
| Pedal edema | 9 (26.5) | 0 (0) |
| Rash | 8 (23.5) | 1 (2.9) |
| Epistaxis | 2 (11.8) | 0 (0) |
| Giddiness | 7 (20.6) | 1 (2.9) |
| Platelet count decreased | 6 (17.6) | 1 (2.9) |
| Hypocalcemia | 5 (14.7) | 0 (0) |
| Fever | 3 (8.9) | 1 (2.9) |
| Hemoptysis | 3 (8.9) | 0 (0) |
